# Good soldiers in implementation: validation of the Implementation Citizenship Behavior Scale and its relation to implementation leadership and intentions to use evidence-based practices

**DOI:** 10.1186/s43058-021-00240-8

**Published:** 2021-12-11

**Authors:** Randi Hovden Borge, Ane-Marthe Solheim Skar, Mathilde Endsjø, Karina M. Egeland

**Affiliations:** Norwegian Centre for Violence and Traumatic Stress Studies (NKVTS), Gullhaugveien 1, 0484 Oslo, Norway

**Keywords:** Evidence-based practice, Implementation citizenship behavior, Implementation leadership, Mental health services

## Abstract

**Background:**

Implementation citizenship behavior (ICB) describes extra-role behaviors performed by employees to support evidence-based practice (EBP) implementation. Such behaviors can be measured using the Implementation Citizenship Behavior Scale (ICBS), which divides ICB into two dimensions, namely helping others and keeping informed. The current study extends the use of the ICBS to a context outside the USA and adds to the literature by investigating how leader-perceived ICB relates to practitioner-perceived implementation leadership and practitioners’ intentions to use EBPs.

**Methods:**

Participants were 42 leaders and 152 practitioners in Norwegian mental health services implementing EBPs for post-traumatic stress disorder. Leaders rated each practitioner on ICB, and each practitioner rated their leader on implementation leadership and reported on their own intentions to use EBPs. The psychometric properties of the ICBS were assessed using confirmatory factor analysis and internal consistency reliabilities. The relationships between ICB, implementation leadership and intentions to use EBPs, were investigated through a series of bivariate correlation analyses and a path analysis of the total scales.

**Results:**

The ICBS showed excellent psychometric properties. The hypothesized two-factor model provided an excellent fit to the data, and both subscales and the total scale were internally reliable. Leader-perceived ICB was positively and significantly correlated with both practitioner-perceived implementation leadership and practitioners’ intentions to use EBPs. Correlations with intentions to use EBPs were stronger for the subscale of keeping informed than for the subscale of helping others.

**Conclusions:**

Results indicated that practitioners who rated their leader higher on implementation leadership received higher ICB ratings from their leader and reported higher intentions to use EBPs. The results provide evidence of a reciprocal social exchange relationship between leaders and practitioners during EBP implementation and a link to an important proximal implementation outcome (i.e., intentions to use EBPs). Results also suggest cultural differences in how ICB is perceived and relates to other phenomena. Scientific and practical implications are discussed.

**Trial registration:**

Retrospectively registered in ClinicalTrials with ID NCT03719651.

Contributions to the literature

- Interest in the role of employees’ citizenship behaviors in evidence-based practice implementation is growing, but is understudied compared to other individual-level phenomena.
- To our knowledge, this study is the first to test the Implementation Citizenship Behavior Scale (ICBS) in a context outside the USA. Our findings indicate that although its psychometric properties are excellent, how citizenship behaviors are perceived and relate to other phenomena might differ between cultural contexts.
- Studies focusing on mechanisms are called upon in the implementation science field. This study introduces a social exchange mechanism to explain the relationship between implementation leadership and employees’ citizenship behaviors.

## Background

Implementing change often involves considerable effort from people at all levels in the organization and may also require employees to go above and beyond their formal job descriptions. A term commonly used to describe such extra-role behaviors is organizational citizenship behavior (OCB), defined as “individual behavior that is discretionary, not directly or explicitly recognized by the formal reward system, and that in the aggregate promotes the effective functioning of the organization” [1]. After its introduction to the field of organizational behavior in the 1980s [2], numerous studies have investigated its antecedents [3], and its link to positive outcomes on both individual and organizational levels is well documented [4].

Interest in OCB has recently expanded from the field of organizational behavior to the study of evidence-based practice (EBP) implementation [5–7]. Even though one study has indicated that OCB might be important for EBP use [8], it has received little attention in relation to EBP implementation specifically. Aiming at capturing the OCBs employees perform to support EBP implementation, Ehrhart and colleagues [9] developed the Implementation Citizenship Behavior Scale (ICBS). The ICBS is a brief and practical 6-item measure containing the two dimensions of OCB the developers considered most relevant for implementation research and practice, namely *helping others* and *keeping informed* [9]. In line with the literature on general OCB, *helping others* captures extra-role behaviors targeted at individuals in the same organization (e.g., assisting colleagues), whereas *keeping informed* concerns extra-role behaviors towards the organization as a whole (e.g., familiarizing oneself with new routines) [3].

The ICBS was first developed and validated in mental health agencies in the USA [9], and its sound psychometric properties were later confirmed within the education sector [10] and in substance use disorder treatment agencies [11], both in the USA. However, to our knowledge, no studies have investigated how the ICBS performs in settings outside the USA. Thus, studies that expand the ICBS to other cultural contexts are needed, especially since scholars in the general OCB literature, have argued that there might be cultural differences in how OCB is perceived, assessed, and understood [12, 13]. For example, Scandinavian workplace culture is arguably known for its low levels of power distance and high levels of work autonomy [14], which might influence both the type and frequency of citizenship behaviors performed by employees, as well as how these behaviors are viewed by leaders. Thus, the first aim of the current study is to evaluate the psychometric properties of an adapted version of the ICBS on a sample of leaders and practitioners in Norwegian mental health services. We do so through confirmatory factor analysis and by examining internal consistency reliabilities of the two subscales (i.e., *helping others* and *keeping informed*) and the total scale.

Our second aim is to expand the growing—but still very limited—literature on how employees’ citizenship behaviors relate to other constructs important for EBP implementation. We do so by investigating the relationship between practitioners’ implementation citizenship behaviors (ICBs)—as perceived by the leader—and practitioners’ perceptions of their leaders’ implementation leadership [15] and their own intentions to use EBPs. According to arguments based on social exchange theory [16], which is an important theoretical foundation for OCB, the leader-follower relationship is key to understanding the dynamics from which OCBs emerge [17]. In short, followers engage in OCB to reciprocate good leader treatment. This theoretical notion is corroborated by solid empirical evidence indicating positive correlations between transformational leadership (i.e., leaders that inspire and motivate their employees) and OCB [18, 19]. Applied to the context of EBP implementation, we can assume that practitioners engage in social exchange relationships with their leader and reciprocate her/his implementation leadership efforts (i.e., leadership behaviors that strategically targets EBP implementation) by engaging in ICBs.

Few studies have investigated the link between practitioners’ ICBs and implementation outcomes. In view of the strong meta-analytic evidence linking general OCB to positive outcomes on both the individual and organizational level [4], as well as one study linking it specifically to EBP use [8], this link seems relevant to investigate further in the context of EBP implementation. Findings from two validation studies of the ICBS in the USA also support this, where the ICBS was found to correlate positively with supervisors’ perceptions of providers’ success in implementing EBPs and provider-reported EBP use [9, 11]. Both studies found stronger correlations with implementation outcomes for *helping others* than for *keeping informed* [9, 11]. Based on all the above, the following hypotheses are included:

*Hypothesis 1: The psychometric properties of the Norwegian version of the ICBS are satisfactory and comparable to those of the original ICBS.*

*Hypothesis 2: Leader-perceived ICB is positively related to practitioner-perceived implementation leadership.*

*Hypothesis 3: Leader-perceived practitioner ICB is positively related to practitioners’ intentions to use EBPs.*

## Methods

### Procedure

The current study uses data collected during a national implementation of EBPs for post-traumatic stress disorder (PTSD) in Norwegian specialized mental health clinics for children and adults [20]. Child and adolescent clinics implemented trauma-focused cognitive behavioral therapy [21], and adult clinics implemented eye movement desensitization and reprocessing therapy [22] and cognitive therapy for post-traumatic stress [23]. An invitation to participate in the implementation project was sent to regional health trusts and clinic leaders. Clinics who were interested in participating were contacted directly by the research team and received further information through e-mail, telephone, and a face-to-face meeting. Clinic leaders informed their staff, and all practitioners received training in evidence-based screening for trauma exposure and posttraumatic stress symptoms. A sub-sample received training in one of the three treatment models. The Leadership and Organizational Change for Implementation (LOCI) [24] was used as an implementation strategy, which entailed that clinic leaders received training and supervision in implementation leadership. The project, including the current study, was approved by the Norwegian Centre for Research Data (No. 60036/3/LH and 60059/3/OOS).

Data used in the current study was collected between July and September 2019, approximately 1 year after EBP training to ensure that leaders and practitioners had enough time to observe each other’s behaviors. Online surveys were administered via email to clinic leaders and practitioners trained in one of the three EBPs. A reminder was sent to those who had not responded 14 and 28 days after the first invitation. Although each clinic as a whole had consented to participate in the national EBP implementation, the individual practitioners’ participation in the survey study was voluntary and based on informed consent. Participants did not receive any compensation for participating.

### Participants

Participants were leaders and practitioners from 43 mental health clinics across Norway. Of the 47 eligible leaders, 42 responded to the online survey (response rate = 89.4 %). Leaders rated a total of 152 practitioners on the ICBS (average of 3.74 practitioners per leader (range = 1-8). Of these practitioners, 109 responded to an online survey, rating their leader’s implementation leadership and their own intentions to use EBPs for PTSD. A large proportion of both samples were female (64.3% of the leaders; 83.6% of the practitioners), which is representative for the larger population of people working in mental health services in Norway [25]. Average age for the leaders were 49.86 (SD = 7.82) and 43.34 (SD = 10.13) for the practitioners. Over half of both samples had an educational background in psychology (52.4% of leaders; 60.5% of practitioners). Average years of work experience in the current occupation were 18.51 (SD = 7.79) for the leaders and 10.86 (SD = 9.22) for the practitioners (Table 1).

**Table 1:** Participant demographics

	Leaders (***N***=42)		Practitioners (***N***=152)
**Gender**			
Women	27 (64.3%)		127 (83.6%)
Men	15 (35.7%)		24 (15.8%)
Missing	0 (0.0%)		1 (0.7%)
**Age**			
Mean (SD)	49.86 (7.82)		43.34 (10.13)
Missing	0 (0%)		115 (14.3%)
**Education**			
Psychology	22 (52.4%)		92 (60.5%)
Medicine	5 (11.9%)		11 (7.2%)
Social worker	8 (19.0%)		17 (11.2%)
Nurse	7 (16.7%)		16 (10.5%)
Other	0 (0%)		11 (7.2%)
Missing	0 (0%)		4 (2.6%)
**Work experience (years)**			
Mean (SD)	18.51 (7.79)		10.86 (9.22)
Missing	1 (2.38%)		5 (3.29%)

### Measures

The Implementation Citizenship Behavior Scale (ICBS) measures the extent to which practitioners exceed expectations at work to support EBP implementation [9]. It consists of six items divided equally across the two dimensions: (1) *helping others* and (2) *keeping informed*. Both subscales showed good internal consistency in the original study (.93 and .91, respectively), as did the total scale (.93). Leaders assessed the frequency with which each practitioner performed the different behaviors related to ICB on a 5-point scale from 0 (“not at all”) to 4 (“frequently, if not always”). The scale was translated into Norwegian with a forward and back translation procedure. Two researchers with experience in implementation science conducted separate forward translations from English to Norwegian. The two versions were compared and combined into one agreed-upon tentative version, which was then back-translated to English by a third member of the research team. The back-translated version was compared to the original version in collaboration with the scale developers before a final version was established. All items were reworded to tailor the measure to the implementation of EBP for PTSD (e.g., “Assisting others to make sure they implement evidence-based practice for PTSD properly” from the *helping others* subscale and “Keeping informed of changes in policies and procedures regarding evidence-based practice for PTSD” from the *keeping informed* subscale*)*.

The implementation leadership scale (ILS) measures unit-level leadership for EBP implementation [15]. It consists of twelve items divided equally across the four leadership dimensions: (1) *proactive* (i.e., anticipating and addressing implementation challenges), (2) *knowledgeable* (i.e., having a deep understanding of the EBP and the implementation), (3) *supportive* (i.e., supporting practitioners’ adoption and use of the EBP), and (4) *perseverant* (i.e., being consistent, unwavering, and responsive to issues and challenges in the EBP implementation) [15]. All scales showed good internal consistency in the original study (ranging from .95 to .98), as well as in a validation study based on data collected during the same national implementation as the current study is based on (ranging from .93 to .97) [26]. Practitioners assessed their leader’s implementation leadership with regards to EBP for PTSD on a 5-point scale from 0 (“not at all”) to 4 (“a very great extent”).

The Measure of Innovation-Specific Implementation Intention (MISII) was designed to measure individual providers’ intentions to implement a specific innovation [27]. It consists of three items that each captures an aspect of intention: plans, desire, and scope. The scale showed good internal consistency in the original study (.90). In the current study, practitioners were asked about their intentions to use EBP for PTSD to (1) screen patients for PTSD (*intend to screen*) and (2) treat patients for PTSD (*intend to treat*). All items were rated on a 5-point scale from 0 (“not at all”) to 4 (“a very great extent”).

### Statistical analyses

Confirmatory factor analysis was performed with MPlus 8 [28] accounting for the nested data structure (TYPE=COMPLEX) and using weighted least square mean and variance adjusted estimation (WLSMV) appropriate for ordered-categorical indicators. Several fit indices were used to determine model fit: comparative fit index (CFI), Tucker-Lewis index (TLI), root mean square error of approximation (RMSEA), and standardized root mean square residual (SRMR). CFI and TLI values above .90 and RMSEA and SRMR values below .08 indicate acceptable model fit [29]. Cronbach’s alphas to assess internal consistency reliabilities for the total scale and the two subscales were calculated with IBM SPSS Statistics 26. To investigate the relationship between the ICBS (total and subscales), the ILS (total and subscales), and the MISII scales, we performed a series of bivariate correlation analyses (Pearson’s *r* in SPSS), as well as a path analysis between the total scales. The path analysis was performed with Mplus 8 [28], where we accounted for the nested data structure in the same way as in the confirmatory factor analysis. We used maximum likelihood estimation with robust standard errors (MLR) appropriate for continuous variables.

## Results

### Confirmatory factor analysis

We examined the ICBS as a model with two correlated first-order factors (i.e., *helping others* and *keeping informed*) measured by three indicators each. Model fit was excellent, as indicated by multiple fit indices (*χ*^2^ (8, *N* = 152) = 8.15, *p* = .42; CFI = 1.00; TLI = 1.00; RMSEA = .011 (90% confidence interval = .000, .096); SRMR = 0.015). All standardized factor loadings were significant and large, ranging from .87 to .97 (Table 2). No modifications of the model were warranted by modification indices. For comparison, we also examined a model with one first-order factor measured by six indicators. Model fit was acceptable (*χ*^2^ (9, *N* = 152) = 25.054, *p* < .01; CFI = .996; TLI = .993; RMSEA = .108 (90% confidence interval = .059, .160); SRMR = .039), but considerably worse than the model with two factors.

**Table 2:** Descriptive statistics, factor loadings, and scale reliabilities for the ICBS

	M	SD	CFA factor loading	Alpha
**ICBS total scale**	3.00	0.85		.95
Helping others	2.82	0.96		.95
Item 1. Responsibilities related to EBP implementation			0.96	
Item 2. Make sure they implement EBP properly			0.97	
Item 3. Helping teach EBP implementation procedures			0.96	
Keeping informed	3.18	0.83		.92
Item 4. Agency communication related to EBP			0.96	
Item 5. Latest news regarding EBP			0.93	
Item 6. Changes in EBP policies and procedures			0.87	

*Note. N*=152. Factor loadings are standardized

### Scale reliability statistics

Internal consistencies of the ICB total scale (.95) and subscales were high (.95 for *helping others* and .92 for *keeping informed*) (Table 2). Mean was a little higher for *keeping informed* (*M* = 3.18, SD = .83) than for *helping others* (*M* = 2.82, SD = .96); mean of the total scale was 3.00 (SD = .85) (Table 2). Subscales were highly correlated with each other (.80, *p* < .01) and with the total scale (.96, *p* < .01; .94, *p* < .01, respectively) (Table 3).

**Table 3:** Descriptive statistics and correlations of the ICBS, ILS, and MIISI scales

	M	SD	1	2	3	4	5	6	7	8	9
1. ICBS total scale	3.00	0.85	--								
2. ICBS: Helping others	2.82	0.96	.96**	--							
3. ICBS: Keeping informed	3.18	0.83	.94**	.80**	--						
4. ILS: Proactive	2.29	0.95	.15	.11	.17†	--					
5. ILS: Knowledgeable	2.29	1.04	.20*	.18†	.20*	.67**	--				
6. ILS: Supportive	3.19	0.74	.19*	.20*	.16	.70**	.58**	--			
7. ILS: Perseverant	2.82	0.88	.13	.08	.16	.76**	.64**	.80**	--		
8. ILS total scale	2.65	0.79	.19*	.16†	.20*	.90**	.84**	.86**	.91**	--	
9. MISII: Intend to screen	3.54	0.80	.26**	.20*	.29**	.13	.04	.13	.08	.10	--
10. MISII: Intend to treat	3.45	0.70	.43**	.39**	.43**	.21*	.15	.24*	.21*	.22*	.52**

*Note. N*=109 for correlations with ILS and MIISI scales. **p < .01; *p < .05; †*p* < .10

### Correlation analyses

The correlation analyses showed all three ICB scales (i.e., total scale, *helping others*, and *keeping informed*) correlated positively with the ILS total scale, as well as with the subscales for knowledgeable and supportive leadership (Table 3). Nearly all correlations were statistically significant. The exceptions were the correlation between *keeping informed* and *supportive,* and the correlations for *helping others* with the ILS total scale and *knowledgeable*, which were both near significant. There were no statistically significant correlations with the subscales for proactive and perseverant leadership, but *keeping informed* correlated near significantly with both. All correlations between the ICB scales and the MISII scales (i.e., *intend to screen* and *intend to treat*) were statistically significant and weak-to-moderate in size (Table 3). Correlations were stronger for *keeping informed* than for *helping others*.

### Path analysis

**Fig. 1:**
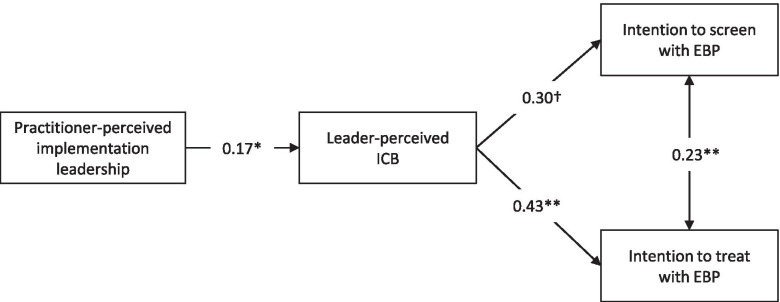
Path analysis. *Note. N*=109. **p < .01; *p < .05; †*p* < .10

A path analysis accounting for the nested data structure showed that practitioner-perceived implementation leadership was significantly related to leader-perceived ICB, which again was significantly related to practitioners’ intentions to use EBP for PTSD to treat patients, and near significantly related to practitioners’ intentions to use EBP for PTSD to screen patients (Fig. 1). Model fit was satisfactory (*χ*^2^ (2, *N* = 109) = 4.03, *p* = .13; CFI = .974; TLI = .922; RMSEA = .096 (90% confidence interval = .000, .234); SRMR = 0.044).

## Discussion

We found that a tailored version of the ICBS showed excellent psychometric properties when tested on a sample of leaders and practitioners in Norwegian mental health services implementing EBPs for PTSD. The two-factor model provided excellent fit to the data, and both subscales and the total scale were internally reliable. These findings support hypothesis one that the psychometric properties would be satisfactory and comparable to those of the original ICBS and contribute to the implementation science literature by expanding the use of the ICBS to a context outside the USA.

Furthermore, our findings indicate a positive relationship between practitioners’ ICBs—as perceived by the leader—and leaders’ implementation leadership—as perceived by the practitioner. This means that practitioners who rated their leader higher on implementation leadership received higher ICB ratings from their leader. Thus, our findings support hypothesis two that there is a positive relationship between leader-perceived practitioner ICBs and practitioner-perceived implementation leadership, and might suggest a reciprocal social exchange relationship between leaders and practitioners during EBP implementation. These findings are in line with meta-analytic evidence from the field of organizational behavior indicating positive correlations between transformational leadership and general OCB [18, 19].

A closer look at the pattern of correlations between the ICBS and the ILS revealed that practitioner ICBs were related to some, but not all, dimensions of implementation leadership. This might suggest that practitioners value some aspects of implementation leadership over others when deciding—consciously or unconsciously—how much extra effort to put into an implementation process. For instance, it might be that practitioners value proactive and perseverant leadership less, at least during the active implementation phase. Alternatively, practitioners might just be more observant of supportive and knowledgeable leadership behaviors. As these findings are based on simple bivariate correlations only, we welcome studies that further investigate how social norms of reciprocity shape behaviors favorable to EBP implementation and potential mechanisms that could explain this relationship. This also includes qualitative research to gain a deeper and more nuanced understanding of these processes (e.g., which specific leadership behaviors practitioners value and why).

We also found a positive relationship between leader-perceived ICBs and practitioners’ own intentions to use EBPs; practitioners who received higher ICB ratings from their leader reported stronger intentions to use EBPs. The relationship was moderate for both subscales, as well as for the total scale. These findings support hypothesis three that there is a positive relationship between leader-perceived practitioner ICBs and practitioners’ intentions to use EBPs, and provide strong evidence for the criterion-related validity of the ICBS with regards to an important proximal implementation outcome [30]. However, the relationship between intention and adoption is not 1:1 [30], and thus, future studies on ICB should strive to include more distal implementation outcomes (e.g., reach) and outcome measures that are not self-report. The latter is particularly interesting to investigate further, as it is not clear from the current study that ICBs come before intentions; one alternative (and equally plausible) interpretation of our findings is that practitioners with higher intentions to use EBPs engage in more ICBs (i.e., a reverse causal relationship).

In contrast to studies validating the ICBS in the US indicating stronger correlations for *helping others* than for *keeping informed* [9, 11], we found instead a tendency for *keeping informed* to be most strongly associated with overall implementation leadership and practitioners’ intentions to use EBPs. Granted that one should be careful to draw any conclusions based on tendencies in small samples, these conflicting findings are still interesting, especially in light of OCB literature drawing attention to potential cultural differences in how OCB is perceived, assessed, and understood [12, 13]. Drawing on Hofstede’s [31] well-known model of national culture, one possible explanation for the difference is Norway’s position on the masculinity/femininity dimension compared to the United States’. Norway is considered among the world’s most “feminine societies” [31], and helping behavior is traditionally viewed as a feminine trait. Thus, it might be that behaviors related to helping others in EBP implementation are expected—or even taken for granted—in Norwegian organizations, and therefore less noticed by leaders, and to a lesser extent viewed as “going above and beyond” to support EBP implementation. Ad hoc analyses support this notion; on average, practitioners were rated significantly higher on *keeping informed* than *helping others* (*t*(151) = 7.85, *p* < .001), although mean scores of both scales were higher in our sample compared to the US samples. Future studies should explore cultural differences in the ICBS further, in terms of both national and workplace culture, by extending its use to other cultural contexts in the Western world, and beyond.

One clear strength of our study is that we use data collected from two different sources (i.e., self-ratings and other-ratings), thus bypassing concerns that our findings were due to common method bias. However, one possible limitation is that our sample is quite small and our data cross-sectional. This made it difficult to control for confounding variables in the analysis. For example, it might be that those practitioners who are already interested in EBPs both intend to use EBPs and are good citizens, and also choose to work at clinics with strong implementation leadership. Given that only 109 out of 152 practitioners responded to the survey, it is also possible that those who responded felt more positively about leadership or were those with greater ICBs than those who did not. An interesting next step for future research would be longitudinal studies in order to address some of these limitations.

Another potential limitation influencing the generalizability of our findings is that data was collected during an active implementation process with strong involvement from the research team. Leaders received training as part of the LOCI strategy and might have been more conscious of their own behavior—as well as more observant of others’—which could have led to inflated correlations. Furthermore, leaders might have rated their practitioners higher on ICBs to give a more favorable impression of their clinic’s implementation efforts; and vice versa, practitioners might have rated their leader higher on implementation leadership in order to make her/him shine during the workshops. However, if this was the case, one would expect high correlations across all subscales, which our findings do not indicate. Nevertheless, our findings might differ from those gained in contexts without external involvement or active implementation. Thus, further research is needed to investigate how the variables in the current study relate in more typical community-care settings.

ICBs have received far less attention in dissemination and implementation (D&I) research and initiatives compared to other practitioner-level phenomena (e.g., attitudes towards EBPs). An important practical implication of the current study is for D&I initiatives to treat practitioners as agents of change during the EBP implementation. Practitioners are likely to be both *influenced by* and *influencing* implementation initiatives; they are not just adopting or not adopting the EBP itself, but also have the potential to be “good soldiers” [1]; the amount of extra effort they put in to support the EBP implementation could have actual consequences for its success. Our findings suggest that supportive and knowledgeable leadership behaviors are particularly important for promoting practitioners’ ICBs. However, it is the practitioners’ *perceptions* of these behaviors that matter. Thus, D&I initiatives should encourage leaders to act supportively and knowledgeably as well as try to make these behaviors visible to the practitioners in their clinic.

## Conclusions

The current study builds on past research that have adapted the concept of OCB to the specifics of EBP implementation [9] and extends the use of the ICBS to a context outside the US. Our findings indicate that although the ICBS worked well in our sample of leaders and practitioners in Norwegian mental health services, how ICB is perceived and relates to other phenomena might differ between cultural contexts. We also found that practitioners who rated their leader higher on implementation leadership received higher ICB ratings from their leader and reported higher intentions to use EBPs. This might indicate that a reciprocal social exchange relationship is in play between leaders and practitioners during EBP implementation. In other words, practitioners look to their leader when deciding how much effort to put in during an EBP implementation. Thus, leaders should be mindful of how their leadership behavior might influence implementation success not only directly, but also indirectly by eliciting certain behaviors in others.

## Data Availability

The dataset used in the current study is available from the corresponding author on reasonable request.

## Abbreviations

OCB: Organizational citizenship behavior
EBP: Evidence-based practice
ICBS: Implementation Citizenship Behavior Scale
ICB: Implementation citizenship behavior
PTSD: Post-traumatic stress disorder
ILS: Implementation leadership scale
MISII: Measure of Innovation-Specific Implementation Intention
